# LncRNA PVT1 promotes the progression of ovarian cancer by activating TGF‐β pathway via miR‐148a‐3p/AGO1 axis

**DOI:** 10.1111/jcmm.16700

**Published:** 2021-07-21

**Authors:** Yuxian Wu, Wenqian Gu, Xiao Han, Zhijun Jin

**Affiliations:** ^1^ Department of Obstetrics and Gynaecology Changzheng Hospital Naval Medical University Shanghai China

**Keywords:** argonaute 1, miR‐148a‐3p, ovarian cancer, PVT1, transforming growth factor‐β

## Abstract

Ovarian cancer is a lethal gynaecologic malignancy with poor diagnosis and prognosis. The long non‐coding RNA plasmacytoma variant translocation1 (PVT1) and argonaute 1 (AGO1) are associated with carcinogenesis and chemoresistance; however, the relationship between PVT1 and AGO1 and the downstream mechanisms in ovarian cancer remains poorly known. PVT1 and AGO1 expression was assessed through RT‐qPCR and Western blotting in both human tissues and cell lines. The viability and proliferation of ovarian cancer cells were determined by CCK‐8 assay and TUNEL assay in vitro and immunohistochemistry in vivo. Cell invasion and migration were investigated through transwell and wound‐healing assays. The roles and mechanisms of AGO1 on cell functions were further probed via gain‐ and loss‐of‐function analysis. We reveal that PVT1 expression was significantly increased in ovarian cancer tissues which is associated with advanced FIGO stage, lymph‐node metastasis, poor survival rate, and high expression of AGO1. PVT1 or AGO1 knockdown significantly reduced the cell viability and increased the cell apoptosis and inhibited ovarian tumour growth and proliferation. Furthermore, we discovered that PVT1 up‐regulated the expression of AGO1 and thus regulated the transforming growth factor‐β (TGF‐β) pathway to promote ovarian cancer progression through sponging miR‐148a‐3p. Additionally, the activation of ERK1/2, smad2 and smad4 is observed to be related to the PVT1/miR‐148a‐3p/AGO1/TGF‐β pathway‐induced cascades. Taken together, the present study reveals that PVT1/miR‐148a/AGO1 axis plays an important role in the progression of ovarian cancer and emphasize the potential as a target of value for ovarian cancer therapy.

**Abbreviations**
AGO1: Argonaute 1
EMT: epithelial‐mesenchymal transition
H&E: haematoxylin and eosin
LncRNA: Long non‐coding RNA
MMP: matrix metalloproteinase
PMSF: phenylmethylsulfonyl fluoride
PVT1: plasmacytoma variant translocation1
TGF‐β: transforming growth factor‐β

## 1 INTRODUCTION

As a lethal gynaecologic malignancy, ovarian cancer often causes high mortality due to its destructive tumour growth and high activity of metastasis. The overall 5‐year survival rate of ovarian cancer patients worldwide is approximately 20%.^1^, ^2^ The symptoms of ovarian cancer are aspecific, and therefore, correct diagnosis is made usually only when widespread metastasis occurs beyond the ovaries. Standard therapy of ovarian cancer includes surgical debulking in combination with chemotherapy with a taxane‐containing platinum doublet.^3^ Although patients may initially respond favourably to this combined therapy, many patients relapse with tumour recurrence due to chemotherapy resistance. Therefore, novel targeted molecular therapies are still urgently needed.

Long non‐coding RNA (LncRNA) is longer than 200 nucleotides which has restricted protein‐coding ability. The main regulatory mechanisms of lncRNAs are as follows: (a) LncRNA competitively adsorbs miRNA by base complementation, which leading to the decrease of miRNA effect on its target genes. (b) LncRNA functions as a precursor of miRNA and promotes miRNA expression and function. (c) LncRNA inhibits or enhances gene transcription through binding chromosomal DNA or recruiting transcription factors. (d) LncRNA activates or inactivates the cell functions through interacting certain epigenetic‐associated proteins, and then regulating its translation.^4^ The lncRNA human plasmacytoma variant translocation1 (PVT1) is located in the protooncogene C‐Myc and human chromosome 8q24, which abundantly expressed in various of tumour tissues and cells, including ovarian cancer.^5^, ^6^ It is reported that PVT1 is related to carcinogenesis and chemoresistance, and the PVT1 expression is elevated in patients with poor prognosis.^7^ In addition, functional inhibition of the PVT1 gene reduced proliferation in ovarian cancer cell lines.^8^ However, the potential effects and mechanisms of PVT1 in carcinogenesis of ovarian cancer remain unknown.

Argonaute 1 (AGO1) is a main constituent of RNA‐induced silencing complexes (RISCs) and predominantly functions in post‐transcriptional mRNA silencing.^9^, ^10^ Several studies suggest that AGO1 shows tissue‐specific high expression and is closely related to the disease progression, including colon cancer,^11^ lung cancer,^12^ breast cancer ^13^ and hepatocellular carcinoma.^14^ Similarly, AGO1 is also overexpressed along the tumour progression in serous ovarian carcinoma.^15^ However, the effects of AGO1 on ovarian cancer, and the relationship between PVT1 and AGO1 are currently unclear and therefore need to be explored.

In this study, we aimed to determine the PVT1 and AGO1 expression in human ovarian cancer tissues and cell lines. Cell lines and xenograft tumours with PVT1 or AGO1 knockdown were used to investigate the effects on cell death and tumour growth. Furthermore, a miRNA intermediary both targeted by PVT1 and binds AGO1 was selected. Finally, the mechanisms of AGO1 of downstream PVT1 axis were determined.

## 2 MATERIALS AND METHODS

### 2.1 Patient samples

Forty‐three ovarian cancer tissue samples were obtained from patients who underwent surgical resection of ovarian. Normal ovarian tissue samples were collected from cervical cancer surgery patients. All tissues were collected from Shanghai Changzheng Hospital from 2014 to 2015. The specimens were frozen immediately in liquid nitrogen and stored at −80°C until further use. Patient follow‐up was performed every 3 months during the first year post‐surgery and then every 6 months thereafter until 5 years after the surgery. Written informed consent was obtained from all patients. This study was performed with the approval of the Ethics Committee of Shanghai Changzheng Hospital.

### 2.2 Cell lines and cell culture

Human ovarian cancer cell lines (SKOV3, A2780, COC1, OVCAR3 and CAOV3) and human ovarian epithelial cell line (IOSE80) were purchased from American type culture collection (Manassas, USA), and HEK293T cell line was maintained in our laboratory. All cell lines were maintained in Dulbecco's modified Eagle's medium (DMEM; Gibco, Grand Island, USA) supplemented with 10% foetal bovine serum (gibco), 100 U/mL penicillin G and 100 μg/mL streptomycin (Thermo Fisher, Waltham, USA) at 37°C in a humidified 5% CO_2_/95% air environment. ERK1/2 inhibitor LY3214996 (10 nmol/L for 1 hours; Selleck, Houston, USA, S8534) or P38 MAPK inhibitor SB203580 (2.5 μmol/L for 8 hours; Selleck, S1076) was added in the medium for further analysis.

### 2.3 Plasmids construction, siRNAs and transfection

DNA segments encoding PVT1 were magnified through PCR from the genomic DNA of SKOV3 cells and loaded into the pCDNA3.0 plasmid. Then, the nucleotide sequences of the construct were verified through DNA sequencing. The hsa‐miR‐148a‐3p mimic and inhibitor were obtained from Ribobio (Guangzhou, China, miR1150109120446‐1‐5 and miR20000243‐1‐5). The sequence of siRNA targeting PVT1#1 was 5′‐GAGCUGCGAGCAAAGAUGU‐3′, #2 was 5′‐ACUUUAAGUGGAGGCUGAAUCAUCU‐3′; the scramble RNA sequence was 5′‐AGAGAGUCAGCAGAUGUCG‐3′. The sequence of siRNA targeting AGO1#1 was 5′‐CCCAGAUACUCCACUAUGA‐3′, #2 was 5′‐ CCCACCCAUUUGAGUUUGA‐3′; the scramble RNA sequence was 5′‐UACAGGAUACUCCACACUC‐3′. Lipofectamine 2000 Transfection Reagent (Invitrogen, Carlsbad, USA, 11668030) was applied to transfect the miR‐148a‐3p mimics, inhibitor, and siRNAs into SKOV3 and HET293T cells. The overexpression vector of AGO1 was transfected through Lipofectamine 3000 Transfection Reagent (Invitrogen, L3000015) followed by the manufacturer's protocols.

### 2.4 RNA extraction and RT‐qPCR

Total RNA was isolated from tissues and cells by Trizol reagent (Invitrogen, 15596026) followed by the manufacturer's protocols. RNA concentration was quantified by a NanoDrop 2000c instrument (Thermo Fisher). cDNAs were generated through reverse transcription with SuperScript II Reverse Transcriptase (Thermo Fisher, Waltham, USA, 18064). Quantitative PCR was carried out utilizing Power SYBR Green PCR Mix (Applied Biosystems, Foster City, USA, 4367659) and mRNA expression was normalized to GAPDH and miRNA level was normalized to U6 using the comparative Ct (2^‐ΔΔCt^) method.^16^ Primer sequences were shown as follows:

PVT1: 5′‐CCTGGTGAAGCATCTGATGCACG‐3′ (forward) and 5′‐GCCAGGCTTTGTGGCACACGC‐3′ (reverse);

AGO1: 5′‐ACTCTACGGTCTGTCCGTTC‐3′ (forward) and 5′‐CCCGCTCAGATGCAATCATTC‐3′ (reverse);

GAPDH: 5′‐GGAGCGAGACCCCACTAACAT‐3′ (forward) and 5′‐ACATACTCAGCACCGGCCTC‐3′ (reverse);

hsa‐miR‐148a‐3p‐RT: 5′‐GTCGTATCCAGTGCAGGGTCCGAGGTATTCGCACTGGATACGACACAAAG‐3′, 5′‐CGGCGGTCAGTGCATCACAGA‐3′ (forward) and 5′‐GTGCAGGGTCCGAGGT‐3′ (reverse);

U6: 5′‐CTCGCTTCGGCAGCACA‐3′ (forward) and 5′‐AAACGCTTCACGAATTTGCGT‐3′ (reverse).

### 2.5 Western blotting

SKOV3 cells were lysed by RIPA buffer (Beyotime; Shanghai, China) with 1 mmol/L phenylmethylsulfonyl fluoride (PMSF), and protein concentrations were determined by BCA Kit (Beyotime). After separating sample proteins through prepared gels (Beyotime) and transferring onto polyvinylidene difluoride membranes (Millipore; Burlington, MA, USA), membranes were blocked and incubated by primary antibodies against relative target proteins overnight at 4°C. The following antibodies and dilutions were used: AGO1 (Cell Signaling, Danvers, USA, 5053; 1:1000 dilution), E‐cadherin (Cell Signaling, 14472; 1:1000 dilution), N‐cadherin (Cell Signaling, 13116; 1:1000 dilution), Vimentin (Cell Signaling, 5741; 1:1000 dilution), Twist (Cell Signaling, 46702; 1:1000 dilution), Snail (Cell Signaling, 3879; 1:1000 dilution), Zeb1 (Absci, Nanjing, China, AB38675; 1:1000 dilution), p‐ERK1/2 at Thr202/Tyr204 (Cell Signaling, 4370; 1:2000 dilution), ERK1/2 (Cell Signaling, 4695; 1:1000 dilution), p‐P38 at Thr180/Tyr182 (Cell Signaling, 4511; 1:1000 dilution), P38 (Cell Signaling, 8690; 1:1000 dilution), p‐Smad2 at Ser245/S250/S255 (Cell Signaling, 3104; 1:1000 dilution), p‐Smad2 at Ser465/Ser467 (Cell Signaling, 18338; 1:1000 dilution), Smad2 (Cell Signaling, 5339; 1:1000 dilution), p‐Smad4 at Thr276 (Absci, AB12637, 1:1000), Smad4 (Absci, AB31017, 1:500) and GAPDH (Cell Signaling, 5174; 1:1000 dilution). After incubating with appropriate horseradish peroxidase‐linked secondary antibodies, membranes were assessed through enhanced chemiluminescence detection kit (Beyotime). Digitized signal was analysed using ImageJ v1.53 software (National Institutes of Health; Bethesda, USA). GAPDH was used as an endogenous control.

### 2.6 Cell function assays

Ovarian cancer cell viability and cell proliferation were verified by CCK‐8 assay (Beyotime, Jiangsu, China, C0037). Briefly, 1 × 10^4^ scramble or si‐PVT1 transfected SKOV3 cells were plated into 96‐well plates (Corning, MA, USA). After 12 hours incubation, 10 μL detecting solution was added into the wells and incubated for 1 hour at 37°C. Absorbance at 450 nm was detected at different time points (0, 12, 24, 36 and 48 hours) by a microplate reader (Synergy4, BioTek; Winooski, VT, USA). For cell proliferation, 1 × 10^3^ cells were plated into 96‐well plates and absorbance at 450 nm was determined at 0, 24, 48, 72 and 96 hours after cell plating. Transwell assays were applied for cell invasion assay.^17^ Briefly, cells were plated into an Matrigel (BD Biosciences, Bedford, USA) pre‐coated 8‐μm pore membrane chamber with serum‐free DMEM. The chemoattractant is the DMEM containing 10% FBS in the bottom chamber. After 48 hours incubation and stained by Giemsa dye (Sigma‐Aldrich, St. Louis, USA, G5637), the migrated cells on the bottom surface were quantified. The wound‐healing analysis was applied to determine the cell migration ability 24 and 48 hours.

### 2.7 TUNEL assay

The apoptotic cells with or without PVT1 or AGO1 knockdown were investigated by In Situ Cell Death Detection Kit (Roche, Basel, Switzerland, 12156792910) following the manufacturer's protocols. Cells were photographed by an Olympus FSX100 microscope (Tokyo, Japan).

### 2.8 Cell cycle analysis

Ovarian cancer cells were cultured to 70%‐80% confluency, then trypsinized and fixed in 75% ice‐cold ethanol overnight, and incubated with 20 µg/mL DNase‐free RNase A at 37°C for 30 minutes. Cells were stained with propidium iodide (PI; 50 µg/mL; Sigma‐Aldrich, P4170) for 15 minutes, and the flow cytometry was performed. Cell cycle profiles were analysed by a MultiCycle software.

### 2.9 Lentiviral production and establishment of stable cell lines

The shRNA targeting PVT1 or AGO1 and their scramble RNA were loaded into the lentiviral plasmid pLKO.1‐puro (Sigma‐Aldrich, SHC016) and cotransfected with the packaging plasmids psPAX2 (addgene, Watertown, USA, 12260) and pMD2.G (addgene, 12259) into HEK293T cells. The concentrated titre of virus suspension was 2 × 10^12^ TU/L. The viral supernatant was harvested 48 hours after the transfection and incubated with SKOV3 cells, and the lentiviral transfection rate in SKOV3 cells was 80%‐90%. Finally, the cells were screened by puromycin (Solarbio, Beijing, China, IP1280) and G418 (Yeasen, Shanghai, China, 60220ES03).

### 2.10 Xenograft experiments

Female athymic BALB/c nude mice (5‐6 weeks old) were obtained from the Jackson laboratories (Bar Harbor, ME, USA). The SKOV3 cells expressing PVT1 shRNA or AGO1 shRNA and their scramble RNA (1 × 10^7^ cells in 200 μL PBS) were mixed with Matrigel (Corning, 354230) and subcutaneously injected into the left and right side of the mouse back, respectively. Mixing with Matrigel can prevent cells from spreading to the blood system as much as possible to affect the tumour growth on the opposite side. Tumour‐bearing mice were euthanized by using overdose of pentobarbital sodium administration (100 mg/kg, iv) if their activity level significantly declined due to tumour burden. Mice were killed 30 days post‐injection to collect and weigh the tumours. Tumour volumes were estimated as the length × width^2^ × 0.5. Animal procedures were approved by the Animal Care and Use Committee of Naval Medical University and in accordance with its ethical standards.

### 2.11 H&E staining and immunohistochemistry

The excised tumours were fixed, dehydrated and embedded in paraffin. Then, samples were sectioned at 7 µm thickness and stained by haematoxylin and eosin (H&E Staining Kit; Abcam, Cambridge, UK, ab245880) following the manufacturer's protocols. For immunohistochemistry assay, sections were deparaffinized, hydrated and microwaved for antigen retrieval. After blocking by 5% bovine serum albumin overnight at 4°C, slides were incubated with anti‐Ki67 primary antibody (Abcam, 15580; at 1:200 dilution) overnight at 4°C. Next day, sections were washed three times and incubated with goat anti‐rabbit horseradish peroxidase‐conjugated secondary antibody (Thermo Fisher, 32260; at 1:50 dilution) for 2 hours at room temperature. After diaminobenzidine staining (Sigma‐Aldrich, D12384), images were captured with an optical microscope and analysed using ImageJ v1.53 software.

### 2.12 miRNA sequencing

Total RNA was isolated through miRNeasy Mini kit (Qiagen, Hilden, Germany, 217004) following the manufacturer's protocols. Small RNA libraries were constructed by TruSeq Small RNA Library Preparation kit (Illumina, San Diego, USA, RS‐200). All libraries were sequenced on Illumina HiSeq4000 generating 50 bp paired‐end reads, which were demultiplexed through the Illumina pipeline CASAVA v1.8. High‐quality reads were gained by trimming adapter sequences, invalid and low‐quality reads from the raw reads. The sequence adjustment was accomplished through the sequences in miRBase v21^18^ database, Rfam 12.0^19^ database, SILVA 115^20^ database and Repbase 20.04^21^ database. The reads were then performed for miRNA analysis through miRDeep2^22^ software. The correlation among samples (si‐PVT1 and scramble groups) was determined through Pearson's correlation coefficient. The difference analysis of miRNA was carried out through A through the edgeR^23^ package in R language. Heat map was generated using TBtools v1.055.

### 2.13 Dual‐luciferase reporter analysis

The dual‐luciferase reporter analysis was performed to investigate the negative roles of PVT1 on miR‐148a‐3p and the negative effects of miR‐148a‐3p on AGO1 as described previously.^24^ The PVT1 sequence including 984‐990 bp and the 3’UTR of AGO1 including 1482‐1488 bp were magnified from HEK293T cells or ovarian cancer SKOV3 cells through PCR and duplicated from the *XhoI* site to the *SalI* site of the pMIRGLO expression plasmid (Promega, Madison, USA, E1330). The miR‐148a‐3p target site‐mutation of PVT1 and AGO1 luciferase reporter plasmids were produced by QuikChange II XL Site‐Directed Mutagenesis Kit (Agilent, Santa Clara, USA, 200521). The sequences of all plasmids were verified through DNA sequencing. The proportion of two luciferases (Firefly luciferase/Renilla luciferase) were recorded.

### 2.14 Statistical analysis

Data were expressed as the mean ± standard error of the mean. Unpaired t test was used to compare quantitative variables between two groups, and one‐way ANOVA followed by Tukey's post hoc test was applied to compare qualitative variables among multiple groups. The overall survival rates were calculated using the Kaplan‐Meier method with the log‐rank test for comparison. *P* < .05 was considered statistically significant. *indicates *P* < .05, **indicates *P* < .01, ***indicates *P* < .001, and ns indicates no significance. Analysis was performed using SPSS 22.0 for Windows (SPSS Inc, Chicago, USA).

## 3 RESULTS

### 3.1 High expression of PVT1 is observed in ovarian cancer

**FIGURE 1:**
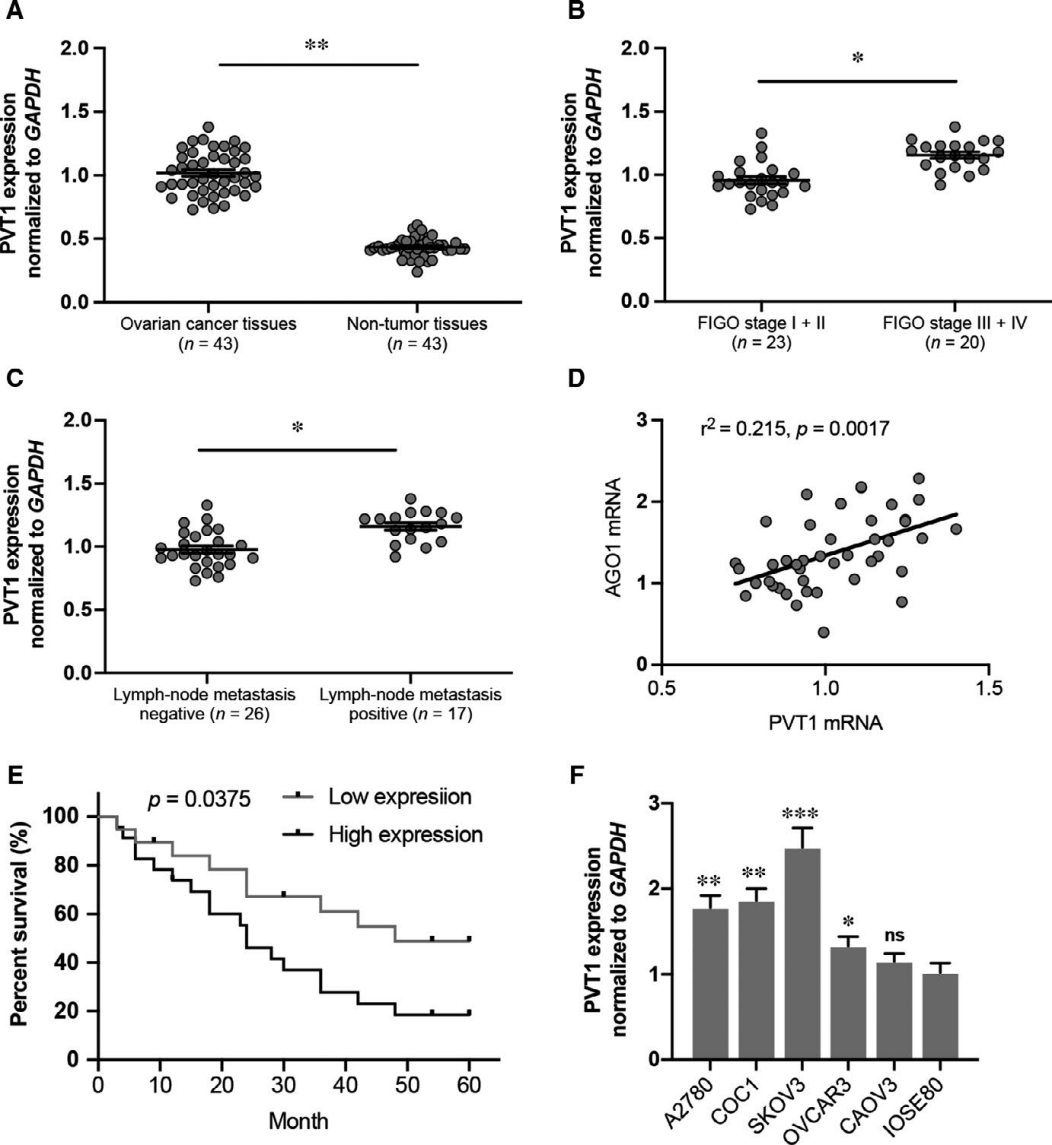
Long non‐coding RNA PVT1 expression was decreased in ovarian cancer. A, PVT1 expression in ovarian cancer tissues and non‐tumour tissues. B, PVT1 expression was up‐regulated in ovarian cancer patients with FIGO stage III‐IV compared to FIGO stage I‐II. C, PVT1 expression was up‐regulated in ovarian cancer patients with lymph‐node metastasis. D, PVT1 expression is significantly positively correlated with AGO1 expression. E, Kaplan‐Meier survival analysis showed that ovarian patients with high PVT1 expression had a poor overall survival. F, PVT1 expression was determined in OC lines by RT‐qPCR. N = 4‐5 independent experiments. *P < .05, **P < .01, ***P < .001 vs. indicated group or IOSE80 group. ns indicates no significance

To explore the role of PVT1 in ovarian cancer progression, we first determined the PVT1 expression in human tissues by RT‐qPCR, and the results showed that a higher expression of PVT1 was detected in ovarian cancer tissues compared to non‐tumour tissues (Figure 1A). Increased PVT1 expression was also observed in ovarian cancer patients with advanced FIGO stage and lymph‐node metastasis (Figure 1B and C). Moreover, correlation analysis revealed that the PVT1 expression was associated with AGO1 expression in ovarian cancer tissues (Figure 1D). Kaplan‐Meier survival analysis showed that ovarian cancer patients with high PVT1 expression had a poor overall survival than patients with low PVT1 expression (Figure 1E). RT‐qPCR revealed that the expression of PVT1 was significantly increased in OC cell lines (A2780, COC1, SKOV3 and OVCAR3, but not CAOV3) compared to human ovarian epithelial cell line (IOSE80) (Figure 1F). These findings suggested that PVT1 is related to AGO1 expression and may promote the ovarian cancer progression. Since the most abundant expression of PVT1 was observed in SKOV3 cells, it was used in the follow‐up mechanical experiment.

### 3.2 Deletion of PVT1 or AGO1 reduces SKOV3 cell viability and promotes cell death

**FIGURE 2:**
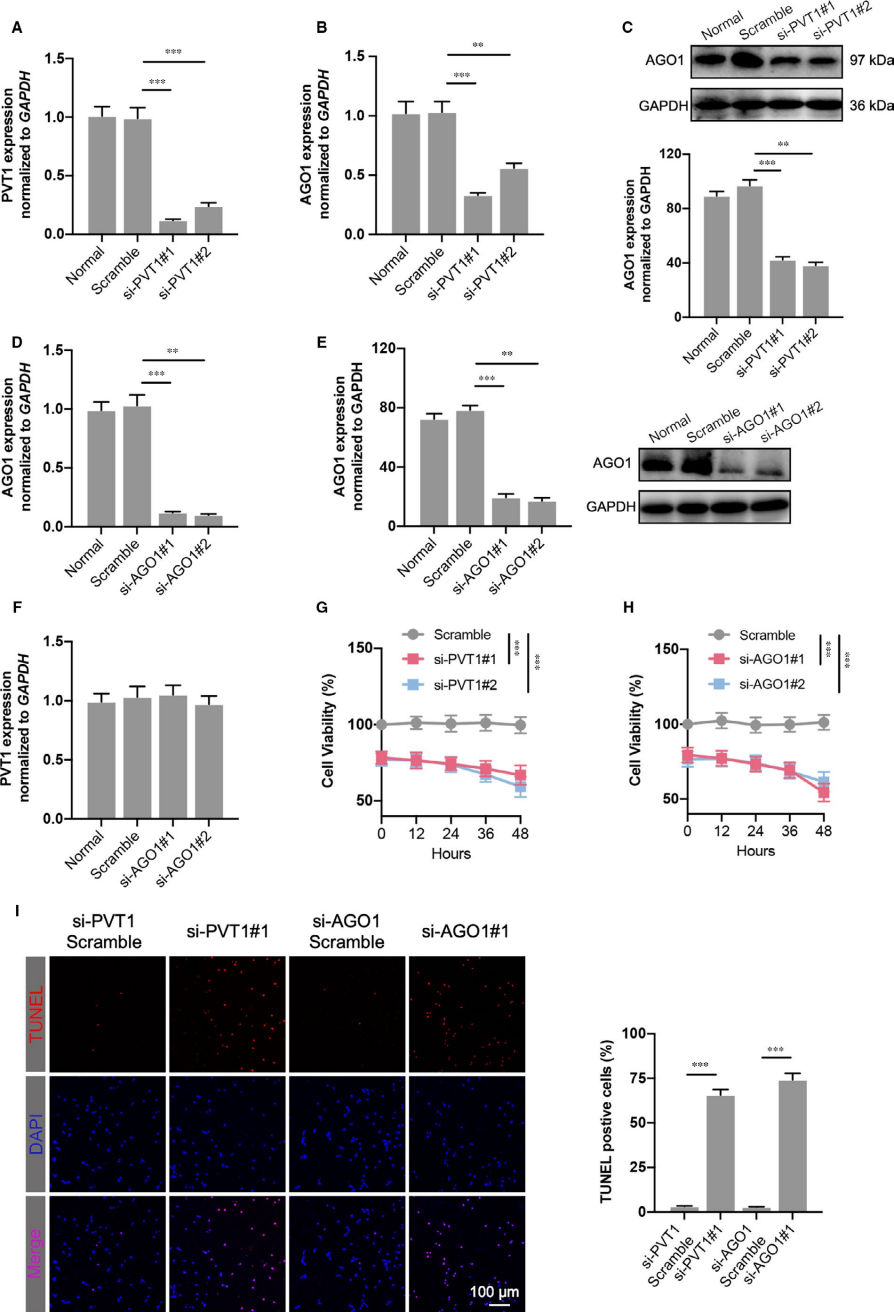
PVT1 or AGO1 depletion decreases ovarian cancer cell viability and increases cell apoptosis. A, PVT1 expression was significantly decreased in SKOV3 ovarian cancer cell line where PVT expression was inhibited after two different siRNA transfection. B and C, AGO1 mRNA level (B) and protein expression (C) were also reduced significantly after siRNA targeting PVT1 transfection. D and E, The validity of siRNAs targeting AGO1 was verified by detecting the mRNA level (D) and protein expression (E) of AGO1 in SKOV3 cells. F, PVT1 level was determined in AGO1‐knockdown SKOV3 cells. G and H, Cell viability was measured using CCK‐8 assay in PVT1‐inhibited (G) and AGO1‐inhibited (H) cells. I, TUNEL assay was performed in transfected SKOV3 cells. The percentage of apoptotic nuclei (red) was calculated by normalizing total nuclei (blue). N = 4‐5 independent experiments. **P < .01, ***P < .001

We constructed two siRNA lentiviral plasmids targeting PVT1 or AGO1. Both si‐PVT1#1 and si‐PVT1#2 transfection in SKOV3 ovarian cancer cells significantly down‐regulated the PVT1 expression (Figure 2A). Importantly, reduced mRNA level and protein expression of AGO1 were also observed, suggesting a secondary down‐regulation of AGO1 (Figure 2B and C). Furthermore, AGO1 inhibition in SKOV3 cells was verified at the nucleic acid and protein level (Figure 2D and E). The PVT1 expression was not affected by AGO1 knockdown (Figure 2F). The cell viability of SKOV3 cells transfected with siRNA targeting PVT1 or AGO1 was significantly decreased compared to the relative scramble group (Figure 2G and h). The cellular apoptosis was also investigated. As shown in Figure 1I, PVT1 or AGO1 deletion dramatically elevated the TUNEL‐positive rate of SKOV3 cells, and no change of the positive rate was observed between the si‐PVT1 and si‐AGO1 groups.

### 3.3 Reduction of tumour growth was observed in tumour‐bearing mice with PVT1 or AGO1 knockdown

**FIGURE 3:**
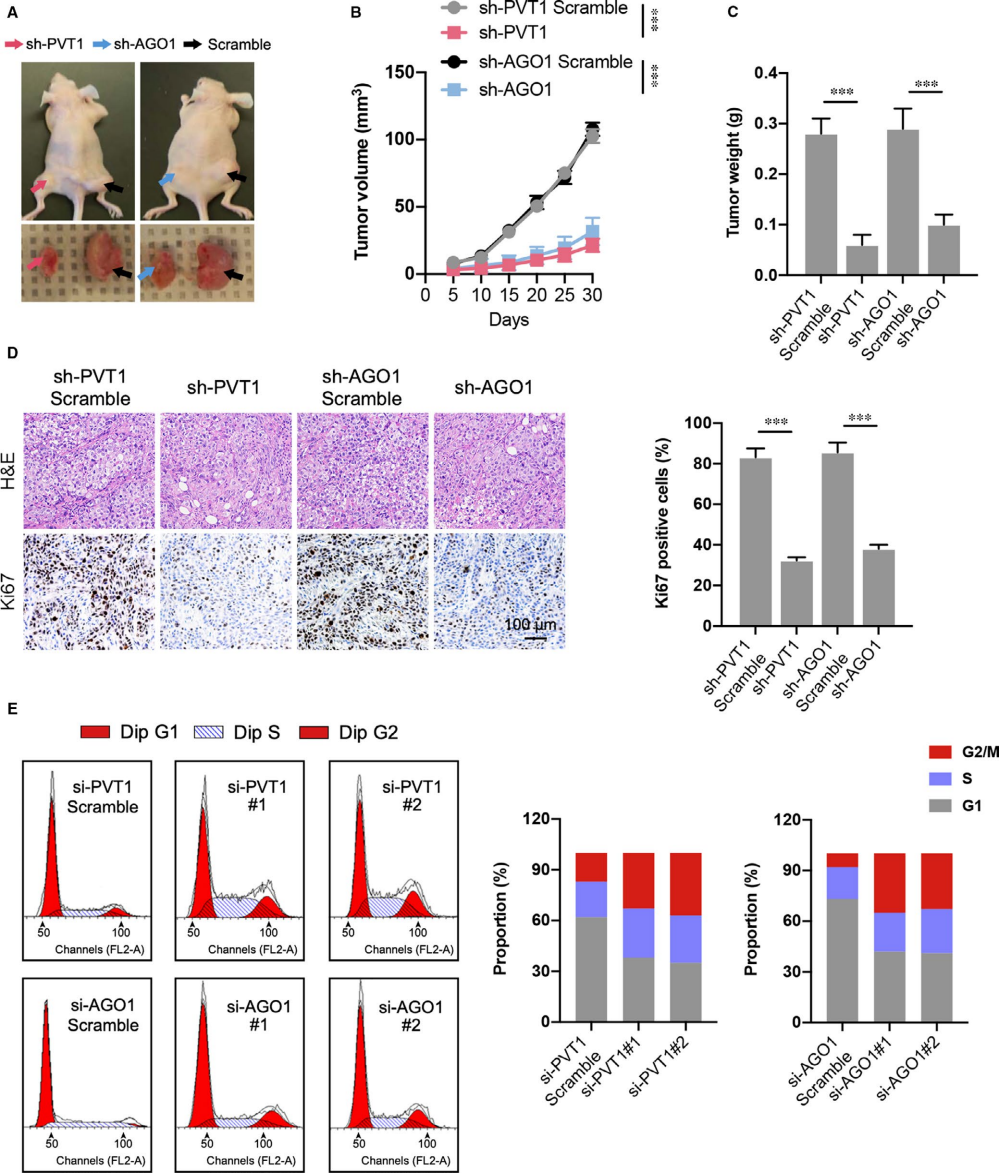
Inhibition of PVT1 or AGO1 alleviates the OC tumour growth in tumour‐bearing mice. A, Representative image of tumour‐bearing mice in each group and tumour excised from the mice were shown. B, Growth curve of tumours in different groups; and the tumour growth status was recorded every 5 days after the cell injection. C, Tumour weight was also recorded in different groups. D, Representative images of H&E staining and immunohistochemical of Ki67 in excised tumour tissues. N = 7 mice per group. E, The cell cycle analysis was performed by flow cytometry in transfected SKOV3 cells. The proportion of each cell cycle phase was calculated. N = 3 independent experiments. ***P < .001

To further determine the tumour suppression of PVT1 or AGO1 on ovarian cancer in vivo, BALB/c nude mouse models bearing subcutaneous ovarian xenograft tumours derived from SKOV3 cells stably inhibiting PVT1 or AGO1 were used. Thirty days after the injection, the tumours showed different growth rates compared to the scramble (Figure 3A). Specifically, the tumour volume curve and tumour weight of sh‐PVT1 or sh‐AGO1 were significantly decreased compared with those in relative scramble (Figure 3B and C). PVT1 or AGO1 inhibition is also responsible for the promoted Ki67 level in excised tumour tissue (Figure 3D). In addition, PVT1 or AGO1 knockdown induced cell cycle arrest at G2/M phase compared to control group (Figure 3E). Collectively, these results reveal that deletion of the high level of PVT1 or AGO1 slowed the ovarian tumour growth in vivo.

### 3.4 PVT1 directly binds miR‐148a‐3p

**FIGURE 4:**
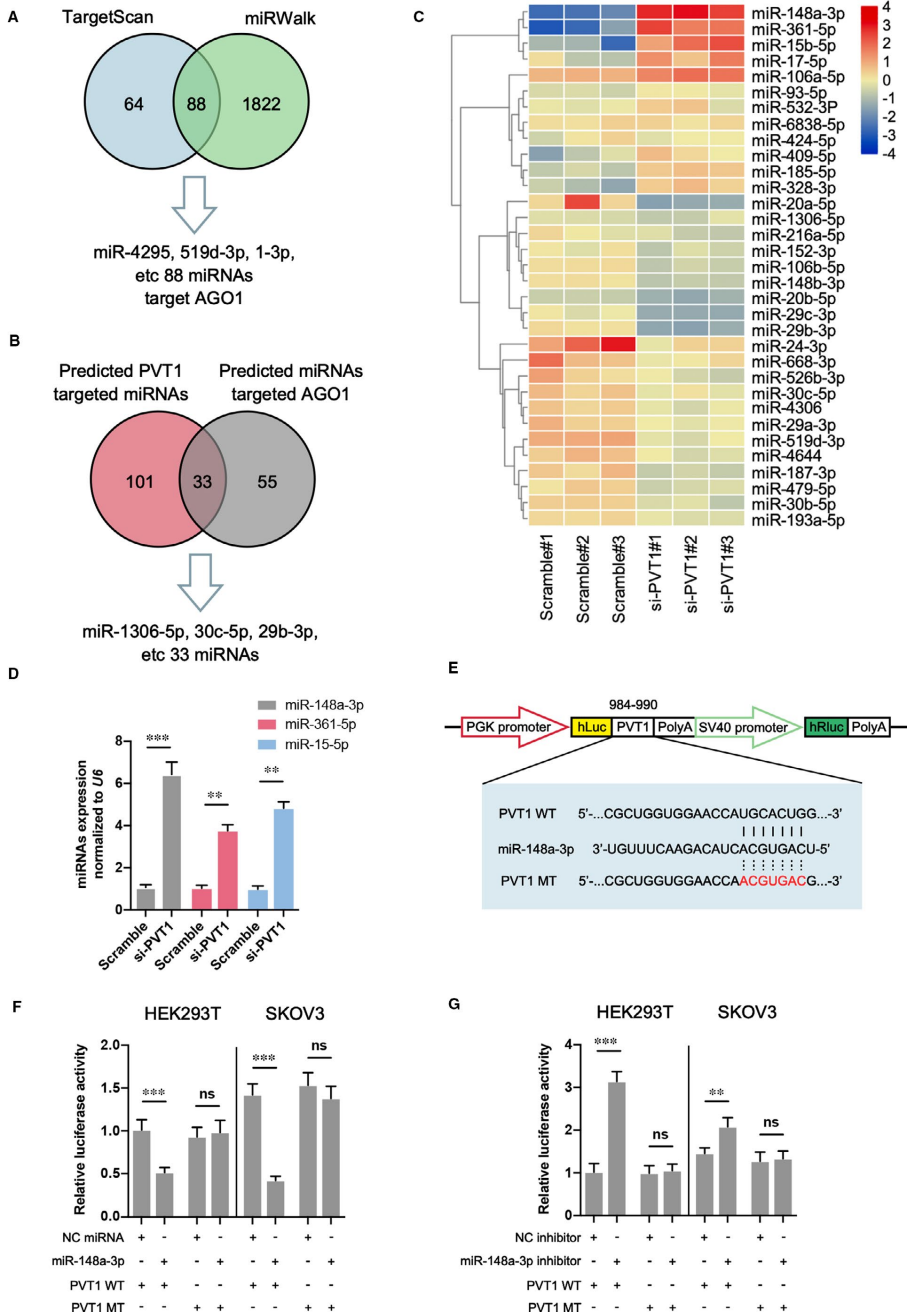
PVT1 directly targets miR‐148a‐3p. A and B, Potential miRNAs that target both AGO1 and PVT1 were predicted by the TargetScan, miRWalk and ENCORI databases. C, The expression of predicted miRNAs target AGO1 and PVT1 was determined through miRNA sequencing in SKOV3 cells transfected with siRNA targeting PVT1 or scramble. Heat map was drawn through TBtools v1.077 software. D, The expression of three most enriched miRNAs (miR‐148a‐3p, miR‐3361‐5p and miR‐15‐5p) was verified in different groups. E, The predicted miR‐148a‐3p binding sequence in PVT1 and the generation of dual‐luciferase reporter plasmids of wild‐type (WT) and mutant (MT) were shown. F and G, Luciferase activity assays were carried out in HER293T or SKOV3 cells cotransfected with miR‐148a‐3p mimic (F) or miR‐148a‐3p inhibitor (G) and PVT1 WT or PVT1 MT. N = 3 independent experiments. **P < .01, ***P < .001. ns indicates no significance

One of the important roles by which lncRNAs function in cell modulation is competitively adsorbing miRNA through base pairing, which leading to the loss‐of‐function of miRNA on target genes.^4^ Therefore, we screened potential miRNA candidates that may be regulated by PVT1. As shown in Figure 4A, 88 miRNAs that target AGO1 were selected by online bioinformatics databases (TargetScan and miRWalk). Then, 33 miRNAs were screened out after the predicted PVT1‐targeted miRNAs were crossed with these 88 miRNAs (Figure 4B). The expression of these selected miRNAs was determined by miRNA sequencing subsequently (Figure 4C). The results suggested that several miRNAs including miR‐148a‐3p, miR‐361‐5p and miR‐15‐5p were up‐regulated after PVT1 inhibition, and miR‐148a‐3p showed the most dramatic elevation, which was further verified by RT‐qPCR results (Figure 4D). Importantly, a significant decrease in miR‐148a‐3p level was also observed in ovarian cancer cell lines and tissues compared to ovarian epithelial cells or non‐tumour tissues (Figure S1A,B). To explore whether PVT1 directly target miR‐148a‐3p, a dual‐luciferase reporter analysis was carried out by a luciferase expression plasmid including the PVT1 fragment containing the presumptive miR‐148a‐3p binding area (Figure 4E). As revealed in Figure 3F, miR‐148a‐3p mimics dramatically declined the activity of the luciferase reporter carrying the PVT1 wild‐type (WT) sequence compared to the controls, and mutation of the miR‐148a‐3p binding area completely abolished these negative roles in HEK293T and SKOV3 cells (Figure 4F). Conversely, miR‐148a‐3p inhibitor up‐regulated the luciferase activity, and the mutation significantly reversed these effects in both cells (Figure 4G). In conclusion, these results demonstrate that PVT1 directly binds and acts on miR‐148a‐3p.

### 3.5 miR‐148a‐3p targets AGO1 and down‐regulates its expression

**FIGURE 5:**
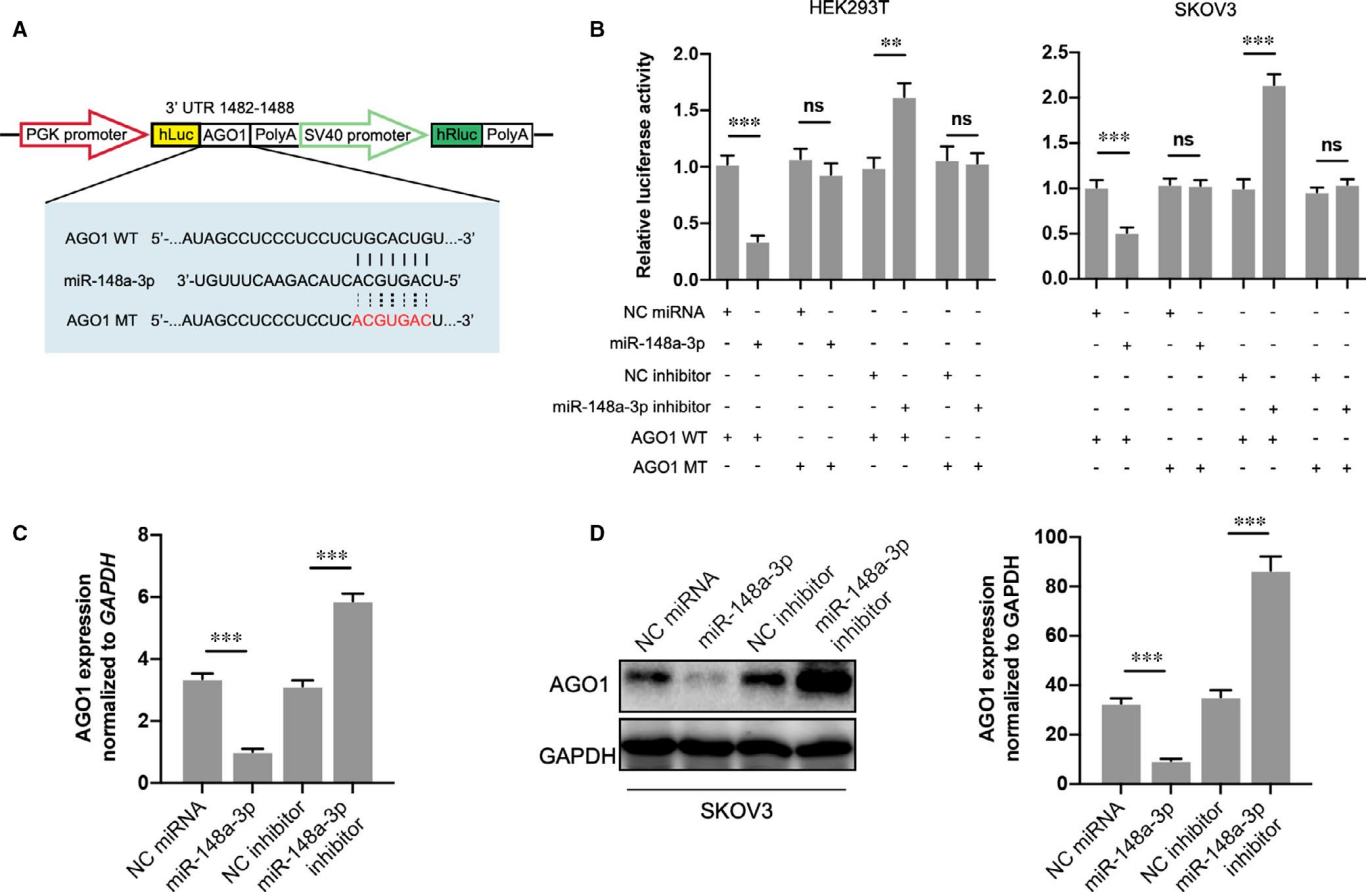
miR‐148a‐3p targets AGO1 and down‐regulates AGO1 expression. A, The predicted miR‐148a‐3p binding sequence in 3’ UTR of AGO1 and the generation of dual‐luciferase reporter plasmids of AGO1 WT and AGO1 MT were shown. B, Luciferase activity assays were carried out in HER293T or SKOV3 cells cotransfected with miR‐148a‐3p mimic or miR‐148a‐3p inhibitor and AGO1 WT or AGO1 MT. C and D, AGO1 mRNA level and protein expression were then investigated in each group through RT‐qPCR and Western blotting. N = 3 independent experiments. **P < .01, ***P < .001. ns indicates no significance

We next investigated the association between miR‐148a‐3p and AGO1 through the luciferase activity analysis in HEK293T and SKOV3 cells transfected with a luciferase vector embodying the predicted miR‐148a‐3p binding sites (Figure 5A). Our data demonstrated that the luciferase activity of the reporter including the WT AGO1 3’UTR sequence was significantly reduced by miR‐148a‐3p treatment, mutation of this sequence abolished the negative roles in HEK293T and SKOV3 cells (Figure 5B). On the contrary, miR‐148a‐3p inhibitor demonstrated an opposite role on luciferase activity. Furthermore, the expression of AGO1 was assessed in SKOV3 cells with miR‐148a‐3p overexpression or knockdown. As shown in Figure 5C and D, miR‐148a‐3p mimics significantly reduced, while its inhibitor up‐regulated the expression of AGO1 at both the mRNA and protein levels. Overall, these findings reveal that the expression of AGO1 was modulated through PVT1/miR‐148a‐3p axis in ovarian cancer.

### 3.6 AGO1 deletion reduces the biological function of ovarian cancer cell

**FIGURE 6:**
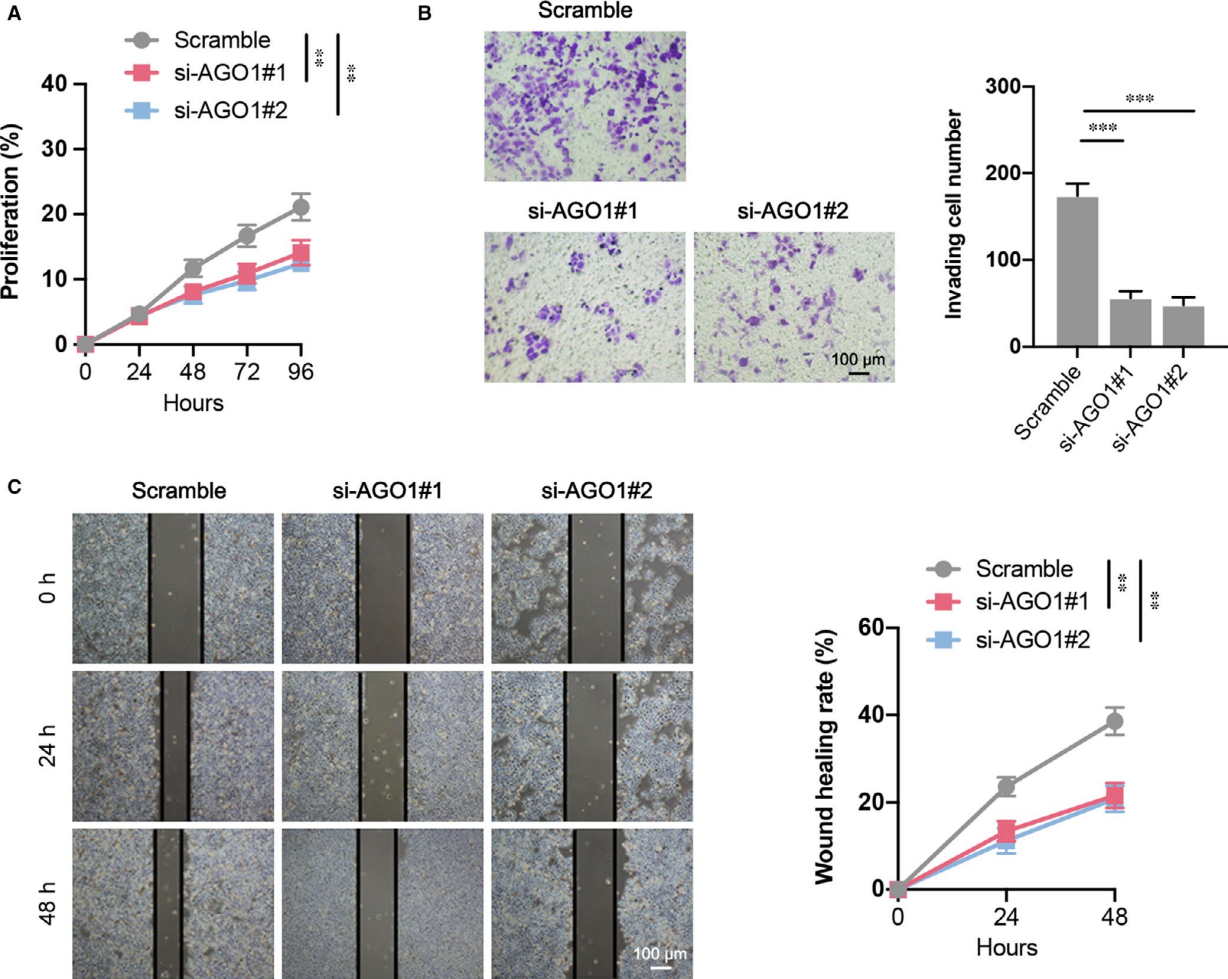
The cell functions of SKOV3 after AGO1 siRNA transfection. A, Cell proliferation of different groups was determined by CCK‐8 assay. B, Cell invasion in each group was determined through a transwell invasion assay. **c** Cell migration of different groups was determined using wound‐healing assay. N = 4‐6 independent experiments. **P < .01, ***P < .001

We next determined the effects of PVT1/miR‐148a‐3p/AGO1 axis on the ovarian cancer cell functions. Down‐regulation of AGO1 by two different siRNAs results in significant decrease of cell proliferation up to 96 hours (Figure 6A). Moreover, the invasion ability of SKOV3 cells with AGO1 knockdown was also inhibited confirmed by transwell invasion assay (Figure 6B). Similarly, declined AGO1 expression in ovarian cancer cell line was accompanied by a greater distance between the two wound fronts compared to scramble group, as determined by the wound‐healing assay (Figure 6C). These data suggest that PVT1/miR‐148a‐3p/AGO1 axis may be an important regulator related to the regulation of cell proliferation and metastasis in ovarian cancer.

### 3.7 AGO1 knockdown suppresses the EMT process and phosphorylation of TGF‐β pathway‐associated proteins

**FIGURE 7:**
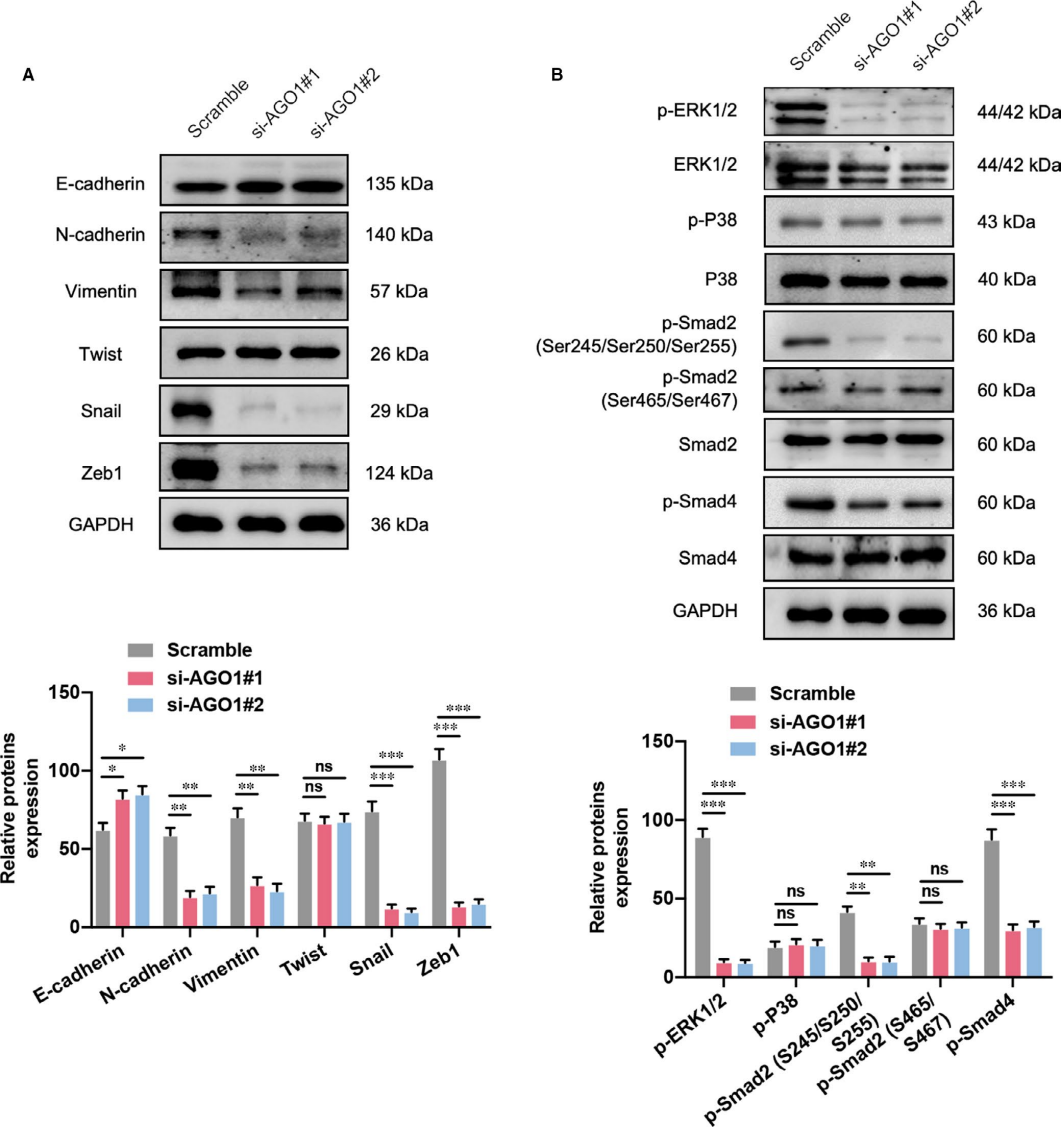
AGO1 promotes OC cell EMT through transforming growth factor‐β (TGF‐β) pathway. A, AGO1 deletion in SKOV3 cells significantly reduced the expression of mesenchymal indicators N‐cadherin and Vimentin, as well as transcription factors Snail and ZEB1; while epithelial indicator E‐cadherin expression was increased, another transcription factor Twist did not show any change. B, The expression of TGF‐β pathway‐related proteins, such as phosphorylated ERK1/2 (p‐ERK1/2 at Thr202/Tyr204), p‐Smad2 (Ser245/S250/S255) and p‐Smad4 (Thr276) was down‐regulated by AGO1 inhibition, and p‐P38 (Thr180/Tyr182), p‐Smad2 (Ser465/Ser467) did not show any change. N = 3 independent experiments. *P < .05, **P < .01, ***P < .001. ns indicates no significance

The epithelial‐mesenchymal transition (EMT) signalling pathways that may be associated with the AGO1 regulatory roles on SKOV3 cell function were then investigated. The Western blotting results suggested that the expression of mesenchymal factors N‐cadherin and Vimentin, and transcription indicators Snail and ZEB1 was significantly decreased in SKOV3 cells with AGO1 knockdown. However, the epithelial indictor E‐cadherin level was slightly up‐regulated, and no change was observed in another transcription indicator Twist (Figure 7A). In addition, as an important signalling pathway related to the regulatory roles on tumour cell activity, we determined the expression of transforming growth factor‐β (TGF‐β) pathway‐associated proteins in AGO1‐depleted cells. As shown in Figure 7B, the phosphorylation of ERK1/2 (Thr202/Tyr204) but not P38 (Thr180/Tyr182) was significantly declined in SKOV3 cells with AGO1 knockdown. Furthermore, the phosphorylation of ERK‐modulated proteins Smad2 (Ser245/S250/S255) and Smad4 (Thr276) was also down‐regulated, and p‐Smad2 (Ser465/Ser467) did not show any change. These data suggest that AGO1 knockdown inhibits the EMT process and TGF‐β pathway in ovarian cancer cell.

### 3.8 ERK1/2 but not P38 involved in the AGO1‐promoted cell proliferation, invasion, and migration

**FIGURE 8:**
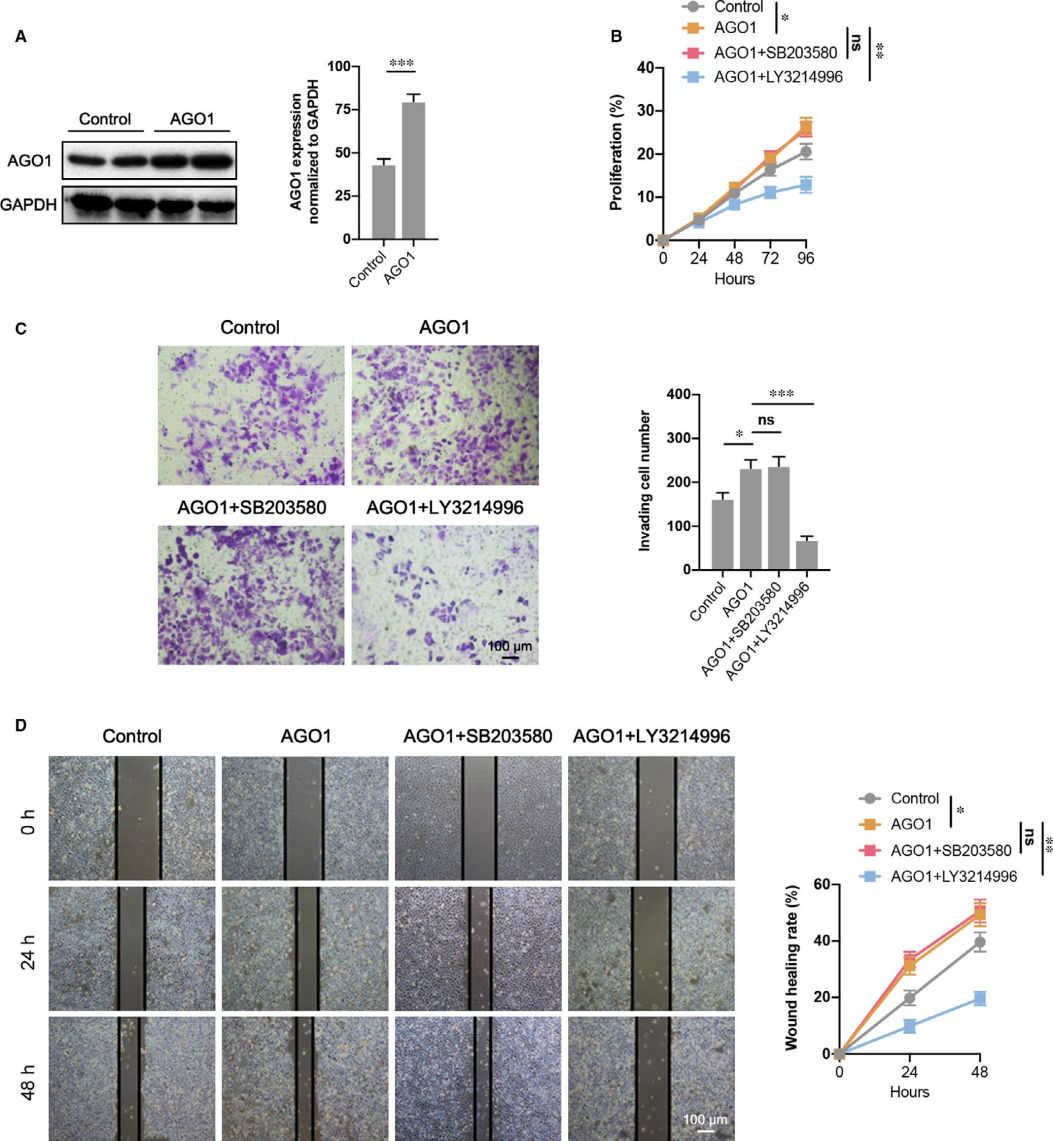
AGO1 promotes cell proliferation, invasion, and migration via ERK1/2 not P38. A, AGO1 overexpression was verified by Western blotting. B, CCK‐8 assay demonstrated a decrease of cell proliferation in AGO1+ERK1/2 inhibitor (LY3214996) transfected SKOV3 cells compared to AGO1 overexpressed cells, while AGO1+P38 inhibitor (SB203580) did not show any change. C, Cell invasion in each group was determined through a transwell invasion assay. D, Cell migration of different groups was determined using wound‐healing assay. N = 4‐6 independent experiments. *P < .05, **P < .01, ***P < .001. ns indicates no significance

To further verify that AGO1 modulated the ovarian cancer cell functions via ERK1/2 not P38, we used the ERK1/2 inhibitor (LY3214996) and P38 inhibitor (SB203580) to inactivate the phosphorylation of ERK1/2 and P38, respectively. The SKOV3 cell line that overexpressed AGO1 was obtained and verified by immunoblot assay (Figure 8A). The cell proliferation was promoted in AGO1 cells compared to control, and LY3214996 treatment significantly declined the proliferation curve. In addition, SB203580 treatment did not affect AGO1 cell proliferation (Figure 8B). Transwell invasion and wound‐healing assays also demonstrated that AGO1 overexpression elevated the cell invasion and migration ability and ERK1/2 but not P38 inhibition reversed these effects in AGO1 cells (Figure 8C and D). Taken together, our data reveal that AGO1 increased the cell proliferation, invasion and migration in SKOV3 cells by regulating ERK1/2.

## 4 DISCUSSION

Nowadays, the late diagnosis and the high rate of chemotherapy resistance of ovarian cancer are contributing to the poor prognosis and great mortality of patients. Recently, research on tumorigenesis‐associated lncRNAs has been gaining increasing attention. Among them, the studies on tumour metastasis‐related lncRNAs are relatively mature. For example, the migration and invasion ability could be increased by overexpressing TP72‐AS1 by positively regulating matrix metalloproteinase (MMP)‐2 and MMP9 in ovarian cancer cells.^25^ In addition, elevated lncRNA EBIC expression increased the migration and invasion ability by targeting Wnt/β‐catenin pathway.^26^ Importantly, Chen et al first reported that MALAT1 played an important role in the distant metastasis of ovarian cancer patients.^27^ Then, MALAT1 suppression inhibited ovarian cancer cell migration and invasion and regulated the MAPK pathway via a variety of mechanisms, including reducing MERK1, ERK1, P38 and JNK phosphorylation.^28^ Similarly, silencing MALAT1 restricted the EMT process and metastasis of ovarian cancer cells via PI3K/Akt pathway inhibition.^29^ Shu et al revealed that lncARSR regulated ZEB1 and ZEB2 to induce the EMT and invasion of ovarian cancer cells by competitively adsorbing miR‐200s.^30^ Furthermore, lncRNA HOXD‐AS1 is reported to increase the EMT by targeting miR‐133a‐3p/Wnt/β‐catenin axis.^31^ Here, we selected the lncRNA PVT1, which is abundantly expressed in ovarian cancer,^8^ as a therapy target for ovarian cancer. Our data suggest that PVT1 promotes ovarian cancer metastasis through PVT1/miR‐148a‐3p/AGO1/TGF‐β axis (Figure S1C).

In our study, we revealed that PVT1 expression was significantly increased in ovarian cancer tissues which is associated with advanced FIGO stage, lymph‐node metastasis, poor survival rate and high expression of AGO1. PVT1 knockdown in SKOV3 significantly reduced the cell viability and promoted the apoptotic rate in vitro. Additionally, PVT1 inhibition also decreased the tumour growth and the proportion of proliferating cells in a xenograft tumour‐bearing mouse model. Importantly, we also investigated the roles of AGO1 in ovarian cancer, a protein highly expressed and closed related to the tumour progression.^15^ Our results also demonstrate that AGO1 knockdown suppress the ovarian cancer progression both in vitro and in vivo. It is reported that miRNA could interact with AGO1 and induce the binding of RISC complex and mRNA targets, results in mRNA degradation or translational inhibition.^32^ miRNA is a genome encoded small RNA deriving form double‐stranded precursor, and function as a post‐transcriptional regulator of target genes responsible for multiple processes, including cell proliferation, differentiation, migration and metabolism.^33^ In fact, miRNA also has been related to various human diseases, such as neurodegenerative diseases and cancer.^34^, ^35^ Specifically, miRNA can function as an oncogene or tumour suppressor in tumour tissues through regulating target genes which are key modulator of tumorigenesis.^34^ All of these studies indicate that miRNA may act as an intermediary bridging PVT1 and AGO1 to regulate the ovarian cancer progression. We therefore focus on miRNA to investigate the potential connection and mechanisms between PVT1 and AGO1. We selected 33 miRNA candidates both targeted by PVT1 and bind AGO1 through online database prediction. Then, we used miRNA sequencing to screen out the 3 highest expression miRNAs in SKOV3 cells with PVT1 knockdown, miR‐148a‐3p, miR‐361‐5p and miR‐15‐5p. Through dual‐luciferase reporter assay, we verified that miR‐148a‐3p can both target PVT1 and AGO1, and exogenous treatment with miR‐148a‐3p mimics dramatically decreased the AGO1 expression. A previous study has detected a low expression level of miR‐148a‐3p in epithelial ovarian cancer tissues which is closely related to the overall survival rate of patients.^36^ In addition, overexpression of miR‐148a‐3p also inhibits the invasion and proliferation ability of SKOV3 cells.^36^ In our study, we reveal the downstream mechanism of miR‐148a‐3p in ovarian cancer is related to the negative regulation of AGO1 through bioinformatics analysis and dual luciferin report experiment, which expands the functional roles of miRNA‐148a‐3p in ovarian cancer.

The TGF‐β pathway is a vital modulator that acts on various physiological and pathological cellular processes. It is well established that TGF‐β can switch its effects from tumour suppressor in normal cells to tumour promoter in advanced cancer cells, facilitating the invasiveness and metastasis of cancer cell.^37^ Furthermore, high level of p‐Smad2 staining in different ovarian tumour types is observed which stimulates the ovarian cancer progression.^38^ The results of a clinical study also showed that a high expression is correlated with poor prognosis in independent advanced high‐grade serous ovarian cancer patients.^39^, ^40^ In our study, overactivation of TGF‐β‐associated proteins is also observed. AGO1 inhibition by siRNAs in SKOV3 significantly reduced the levels of EMT‐related proteins such as N‐cadherin, Vimentin, Snail and Zeb1 and abolished the activation of TGF‐β‐related proteins including ERK1/2, Smad2 (Ser245/Ser250/Ser255) and Smad4, but not P38 MAPK and Smad2 (Ser465/Ser467). As Ser245 of Smad2 is reported to be specifically phosphorylated by ERK and JNK in vascular endothelial cells and involved in endothelial dysfunction,^41^ our data expansively demonstrate that the phosphorylation sites of Ser245, Ser250 and Ser255 on Smad2 is involved in the ERK‐induced cascades at least in ovarian cancer cells with AGO1 knockdown. To further confirm the roles of ERK1/2 downstream of AGO1 in SKOV3 cells, ERK inhibitor LY3214996 or P38 inhibitor SB203580 was treated to investigate the changes of ovarian cancer cell functions. Our loss‐of‐function analysis indicates that LY3214996 but not SB203580 dramatically reduced the cell proliferation, invasion and migration in AGO1‐overexpressed SKOV3 cells, which establishes the important roles of ERK1/2 downstream of the PVT1/miR‐148a‐3p/AGO1 axis.

Also, this study has some limitations. For example, we studied the TGF‐β pathway downstream the PVT1/miR‐148a‐3p/AGO1 signalling; however, there may be other pathways that are also participate in the regulatory roles of this axis on ovarian cancer cells, which are also worth studying. Additionally, in our study, we only demonstrate the important regulatory effects of PVT1/miR‐148a‐3p/AGO1 axis in SKOV3 cell line, and other ovarian cancer cell lines such as A2780, COC1, OVCAR3 and CAOV3 should also be further investigated.

In conclusion, our findings provide insight into PVT1/miR‐148a‐3p/AGO1 axis to be a novel therapy target for human ovarian cancer.

## CONFLICTS OF INTEREST

The authors Yuxian Wu, Wenqian Gu, Xiao Han and Zhijun Jin declare that they have no conflicts of interest that might be relevant to the contents of this manuscript.

## AUTHORS’ CONTRIBUTIONS

ZJ: Conception and design of the study, and manuscript writing; YW and WG: in vivo experiments and data analysis. XH: in vitro experiments and result interpretation. All authors read and approved the final manuscript.

## ETHICS APPROVAL

Animal procedures were approved by the Animal Care and Use Committee of Naval Medical University and in accordance with its ethical standards.

## Supporting information

Fig S1

Supplementary Material
