# Supplementary figures and images for: LncRNA PVT1 promotes the progression of ovarian cancer by activating TGF‐β pathway via miR‐148a‐3p/AGO1 axis
Source: J Cell Mol Med. 2021 Jul 21;25(17):8229–43. doi: 10.1111/jcmm.16700 (PMC8419181; doi:10.1111/jcmm.16700)

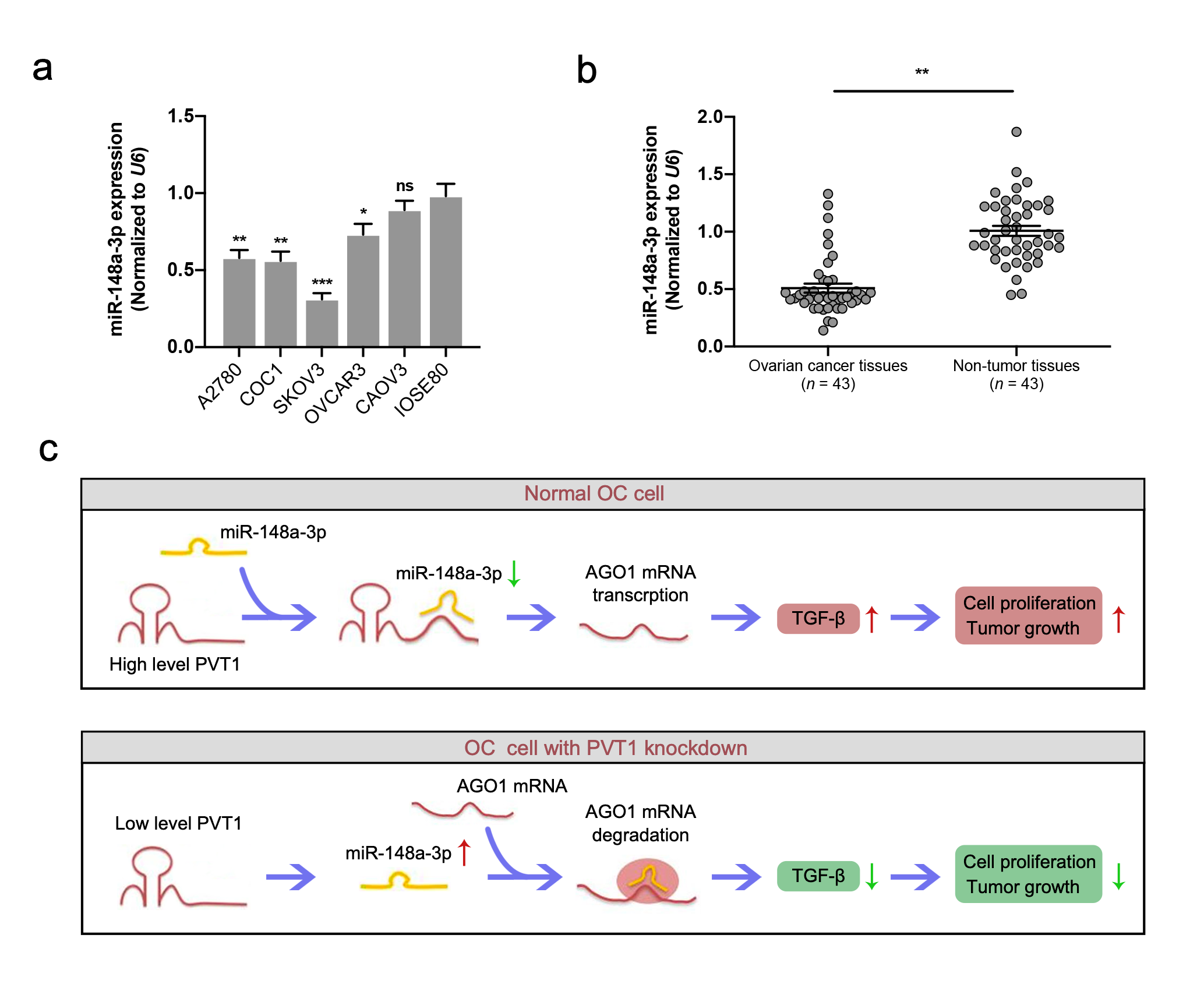

Supplement: Supplementary file 1 — Fig S1 [file JCMM-25-8229-s002.tif]
