# Supplementary material for: LncRNA PVT1 promotes the progression of ovarian cancer by activating TGF‐β pathway via miR‐148a‐3p/AGO1 axis
Source: J Cell Mol Med. 2021 Jul 21;25(17):8229–43. doi: 10.1111/jcmm.16700 (PMC8419181; doi:10.1111/jcmm.16700)
Supplement: Supplementary file 2 — Supplementary Material [file JCMM-25-8229-s001.docx]

**Supplementary figure legends**

Fig.S1 miR-148a-3p expression in ovarian cancer tissues and cell lines. **a** miR-148a-3p level was determined in OC lines and human ovarian epithelial cell line by RT-qPCR. N = 4-5 independent experiments. **b** miR-148a-3p expression in ovarian cancer tissues and non-tumor tissues. **c** A schematic graph for PVT1/miR-148a-3p/AGO1/TGF-β axis in normal ovarian cancer cell and PVT1-inhibited ovarian cancer cell. *p < 0.05, **p < 0.01, ***p < 0.001 vs. IOSE80 group or indicated group. ns indicates no significance; green arrows indicate decrease or inactivation; red arrows indicate promotion or activation.
